# Supplementary material for: Host subversion of bacterial metallophore usage drives copper intoxication
Source: mBio. 2023 Sep 22;14(5):e01350-23. doi: 10.1128/mbio.01350-23 (PMC10653882; doi:10.1128/mbio.01350-23)
Supplement: Supplemental Figures — Figures S1 to S4. [file mbio.01350-23-s0001.docx]

**
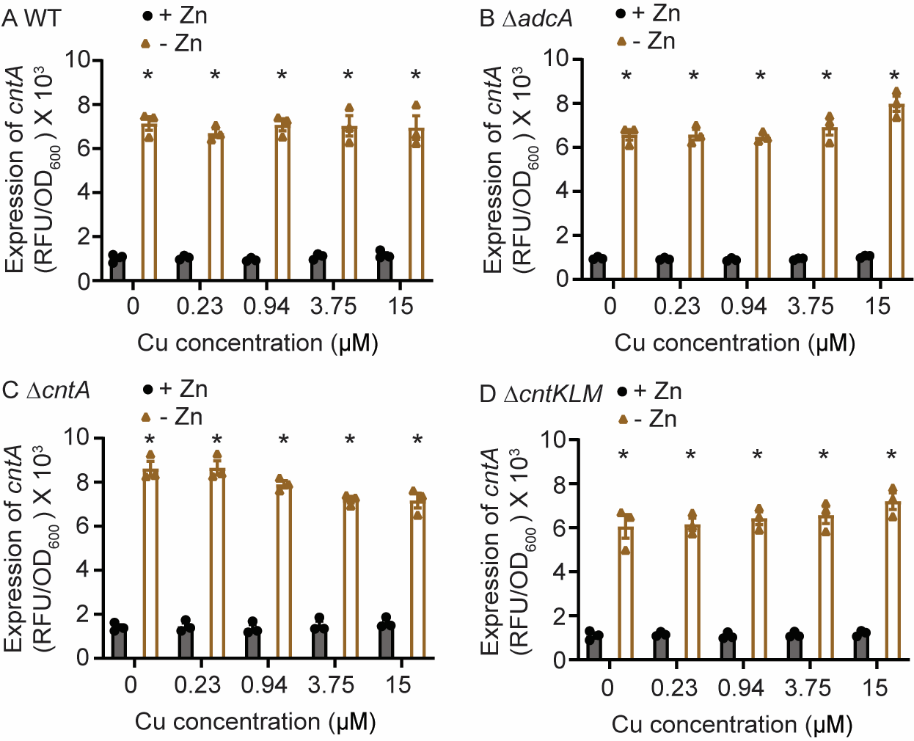
**

**Supplemental Figure 1: Zn limitation induces expression of the Cnt system in a Cu-containing medium.** (A-D) *S. aureus* Newman wild type and the indicated strains containing P*_cnt_*-YFP reporter were grown in NRPMI containing a range of CuSO_4_ concentrations in the presence or absence of 10 μM ZnSO_4_ as specified. The expression of *cnt* was assessed by measuring fluorescence at T = 6 hr. * = p ≤ 0.05 relative to the same strain with Zn via two-way ANOVA with Sidak’s posttest. n ≥ 3. Error bars = SEM.

**
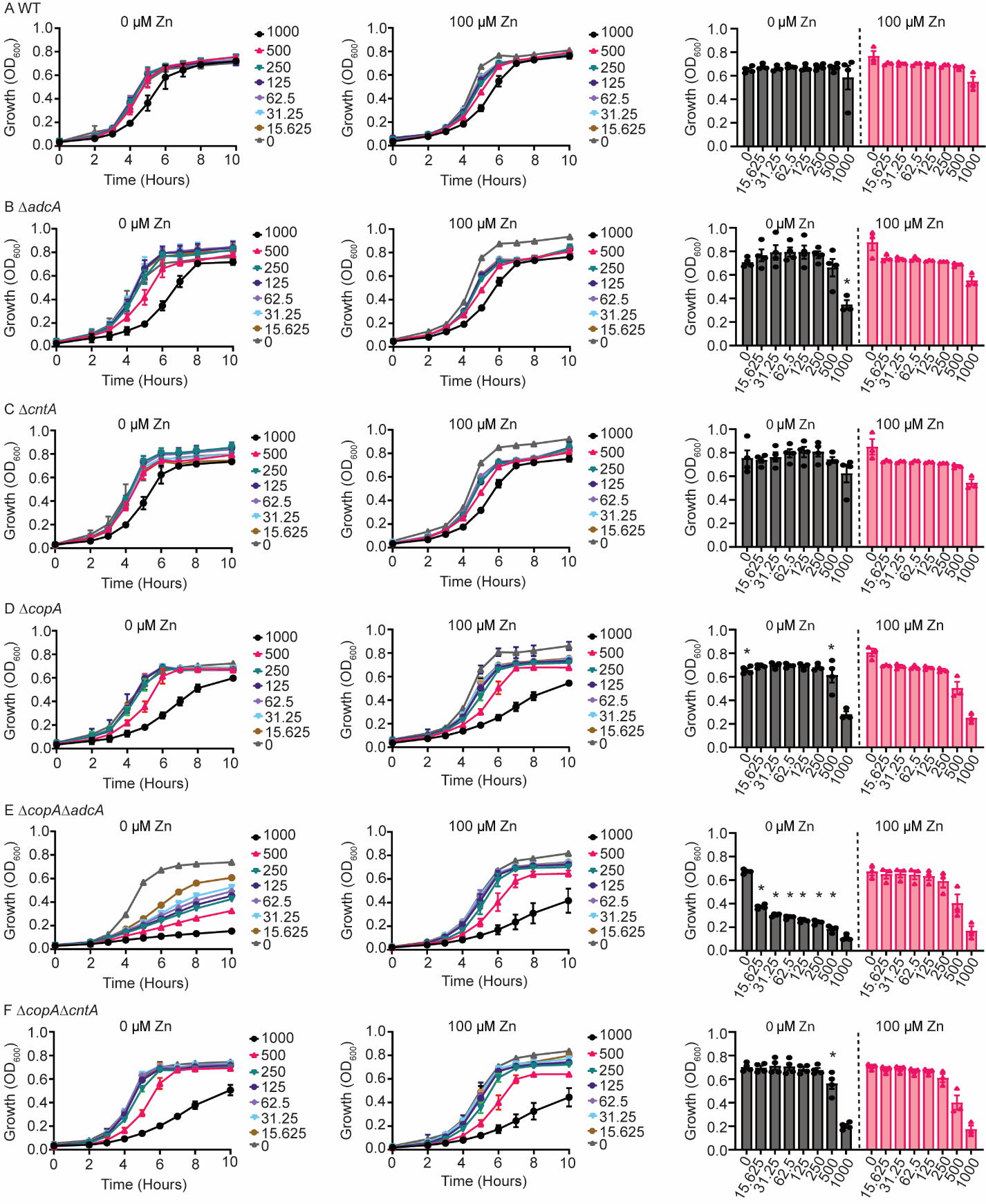
Supplemental Figure 2: The Cnt system increases the susceptibility of *S. aureus* to Cu poisoning.** (A-F) *S. aureus* Newman wild type and the indicated mutants were grown in the absence or presence of 100 μM ZnSO_4_ in NRPMI medium in the presence of various concentrations of CuSO_4_. Growth was assessed by measuring OD_600_ over time. Growth at 6 hours is shown and statistical analysis is present in the bar graphs. * = p ≤ 0.05 relative to the strain cultured under the same Cu concentration with Zn via two-way ANOVA with Sidak's multiple comparisons test. Relevant statistical differences are being shown. n ≥ 3. Error bars indicate SEM.

**
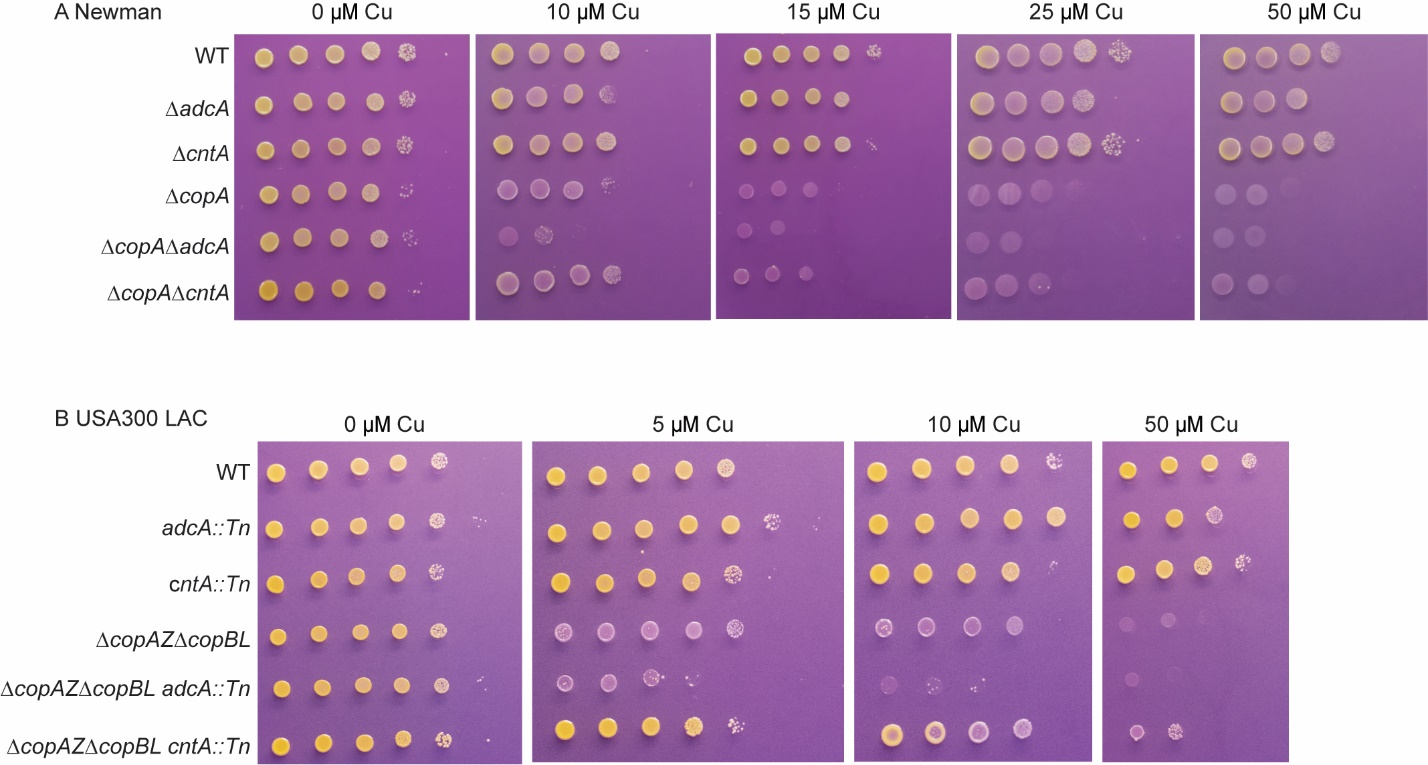
**

**Supplemental Figure 3: Reliance on the Cnt system increases Cu sensitivity in both Newman and USA300 LAC strain backgrounds.** *S. aureus* (A) Newman and (B) USA300 LAC wild type strains and the indicated mutants were cultured in Zn-limited NRPMI and then spot plated onto plates with or without Cu as specified. Representative images of the spot plates are shown here.

**
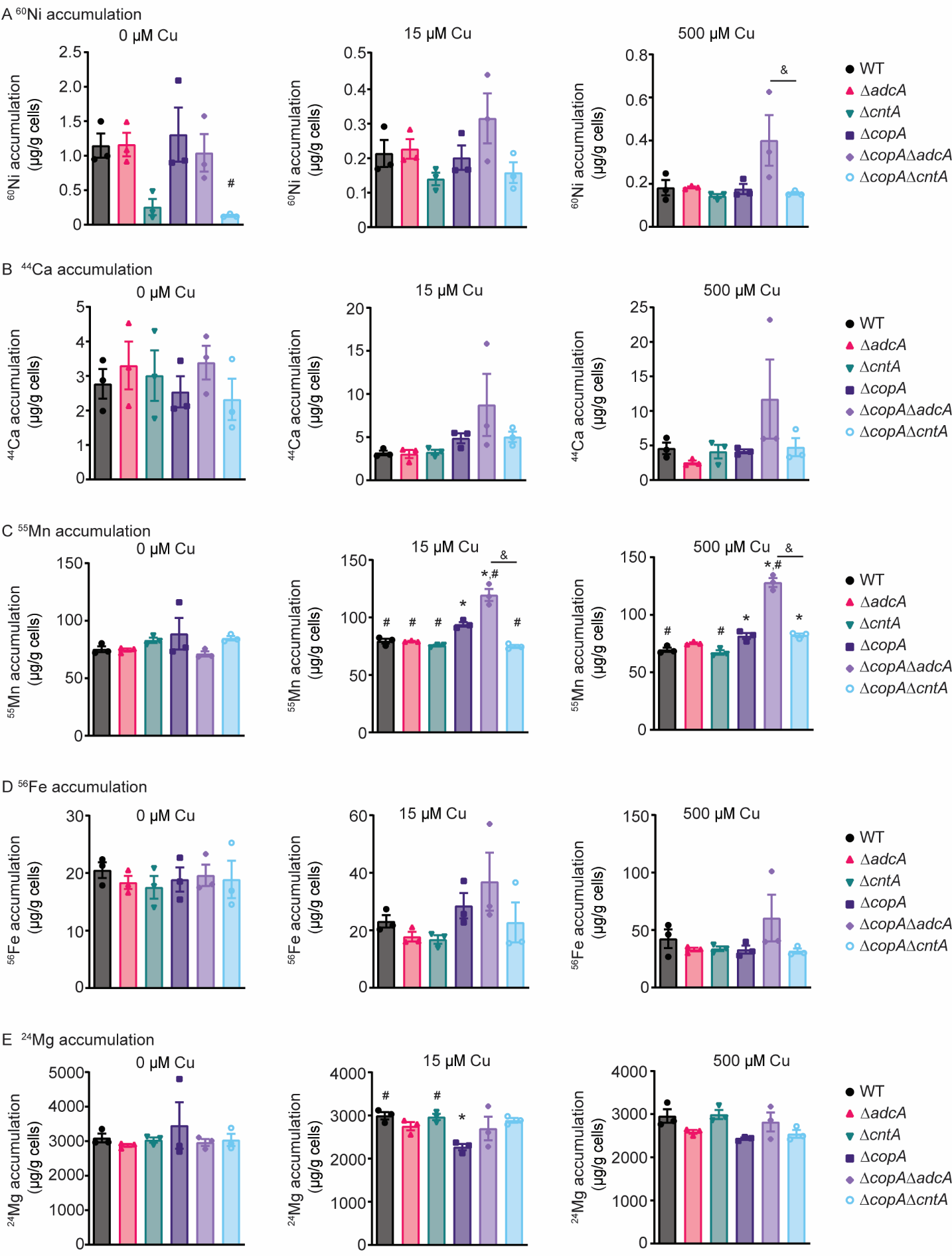
**

**Supplemental Figure 4: Metal accumulation in *S. aureus* when grown in the presence of Cu.** *S. aureus* Newman wild type and the indicated mutants were grown in Zn-limited medium supplemented with 0 μM, 15 μM, and 500 μM CuSO_4_ and cellular (A) nickel (^60^Ni), (B) calcium (^44^Ca), (C) manganese (^55^Mn), (D) iron (^56^Fe) and (E) magnesium (^24^Mg) were assessed using ICP-MS. * = p < 0.05 via one-way ANOVA relative to wild type bacteria using Tukey’s posttest. # = p < 0.05 via one-way ANOVA relative to Δ*copA* using Tukey’s posttest. & = p < 0.05 via one-way ANOVA for the indicated comparison via Tukey’s posttest. n = 3 biological replicates. Error bars indicate SEM.
